# Author Correction: Bottom-up synthesis of chiral covalent organic frameworks and their bound capillaries for chiral separation

**DOI:** 10.1038/s41467-019-08947-y

**Published:** 2019-03-01

**Authors:** Hai-Long Qian, Cheng-Xiong Yang, Xiu-Ping Yan

**Affiliations:** 1 College of Chemistry, Research Center for Analytical Sciences, State Key Laboratory of Medicinal Chemical Biology, Tianjin Key Laboratory of Molecular Recognition and Biosensing, Nankai University, 94Weijin Road, Tianjin, 300071 China; 2 Collaborative Innovation Center of Chemical Science and Engineering (Tianjin), 94 Weijin Road, Tianjin, 300071 China

Correction to: *Nature Communications*; 10.1038/ncomms12104; published online 12 July 2016

This Article contains an error in Fig. 1, in which the structures of ‘(+)-Ac-L-Ta’ and ‘CTp’ are drawn incorrectly. The correct version of Fig. 1 is shown below. The error has not been fixed in the original PDF and HTML versions of the Article.

**Figure 1:**
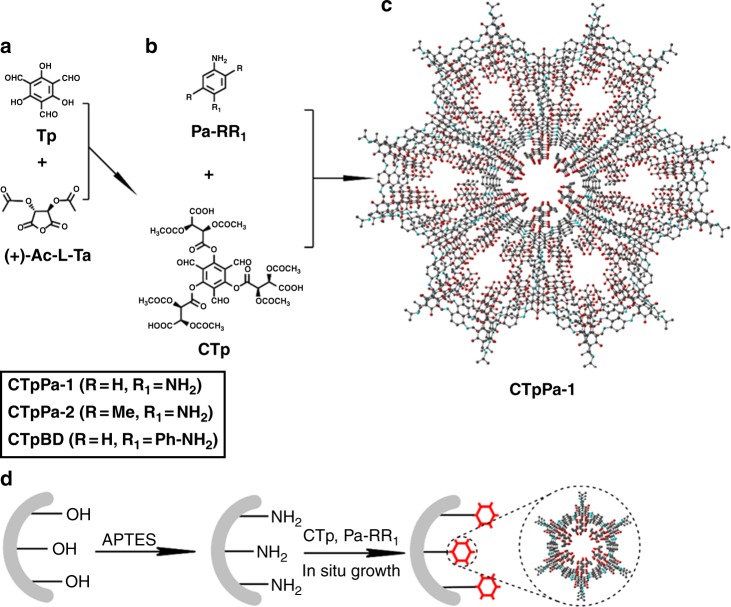
Fig. 1

