# Supplementary material for: Bottom-up synthesis of chiral covalent organic frameworks and their bound capillaries for chiral separation
Source: Nat Commun. 2016 Jul 12;7:12104. doi: 10.1038/ncomms12104 (PMC4945876; doi:10.1038/ncomms12104)
Supplement: Supplementary Information — Supplementary Figures 1-55, Supplementary Tables 1-8, Supplementary Methods and Supplementary References [file ncomms12104-s1.pdf]

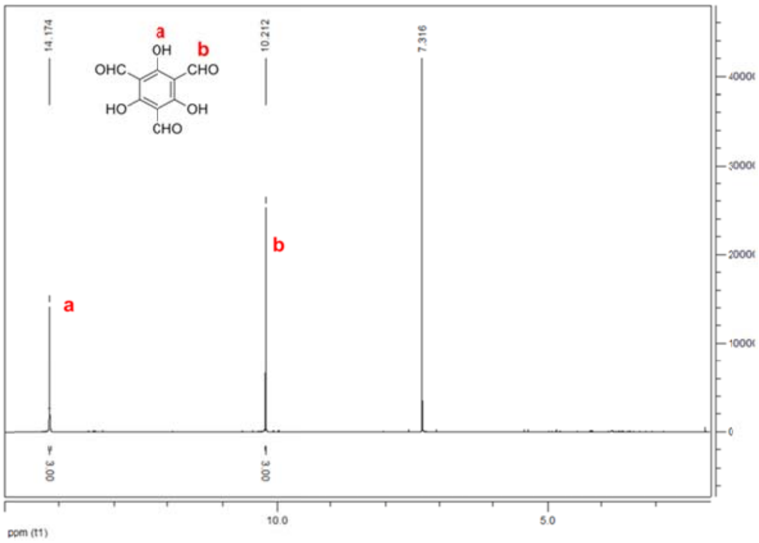

Supplementary Figure 1. ^{1}H NMR spectra of Tp.

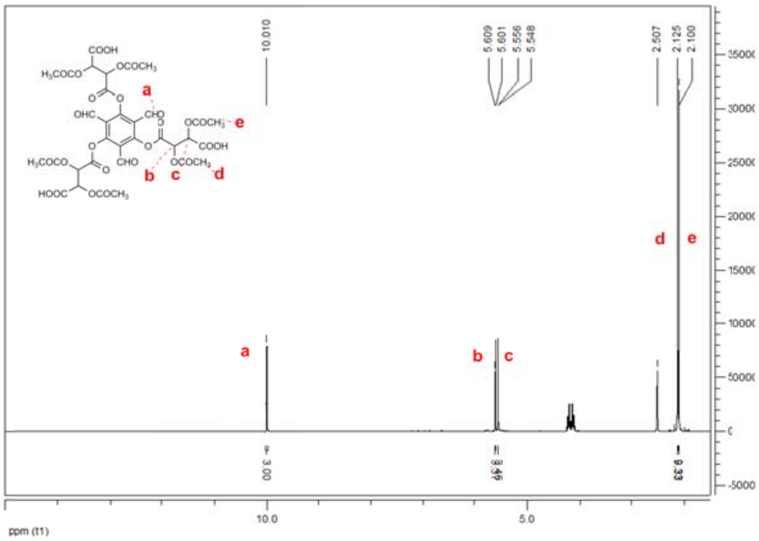

Supplementary Figure 2. ^{1}H NMR spectra of CTp.

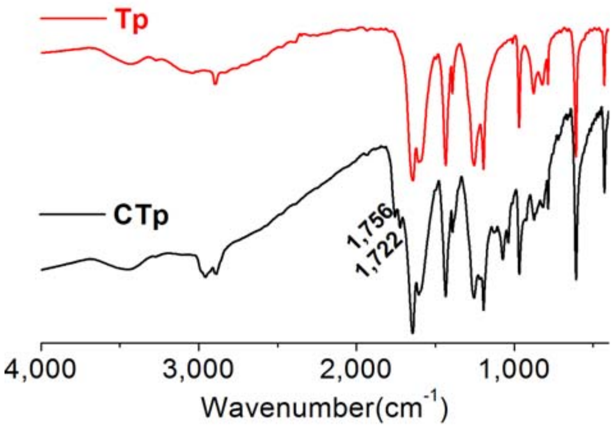

Supplementary Figure 3. FT-IR spectra of Tp (red curve) and CTp (black curve).

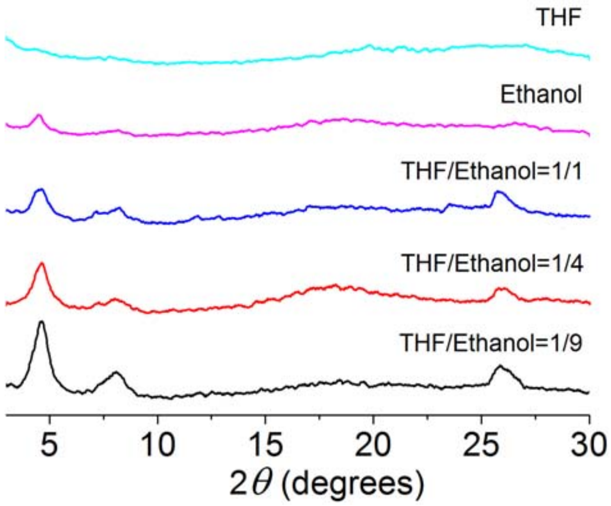

Supplementary Figure 4. Effect of solvent on the PXRD pattern of the CTpPa-1.

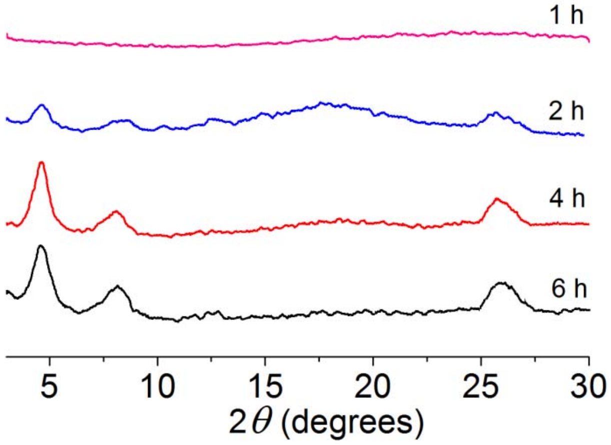

Supplementary Figure 5. Effect of reaction time on the PXRD pattern of CTpPa-1.

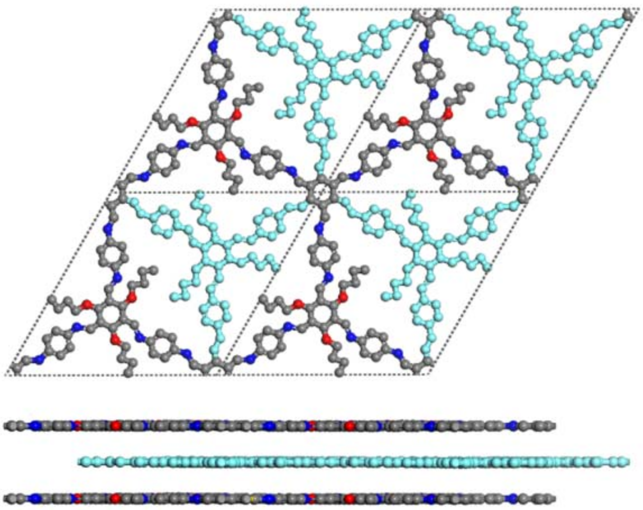

Supplementary Figure 6. The architecture of CTpPa-1 with a staggered arrangement.

H atoms and some atoms on the chiral ligand are omitted for clarity.

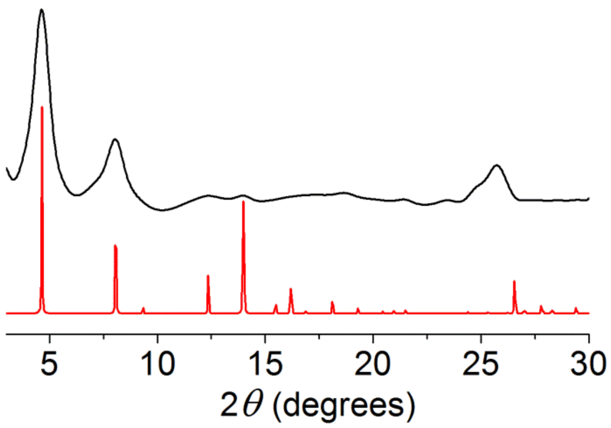

Supplementary Figure 7. Experimental (black) and calculated (red) PXRD patterns of CTpPa-1 with the staggered stacking structure.

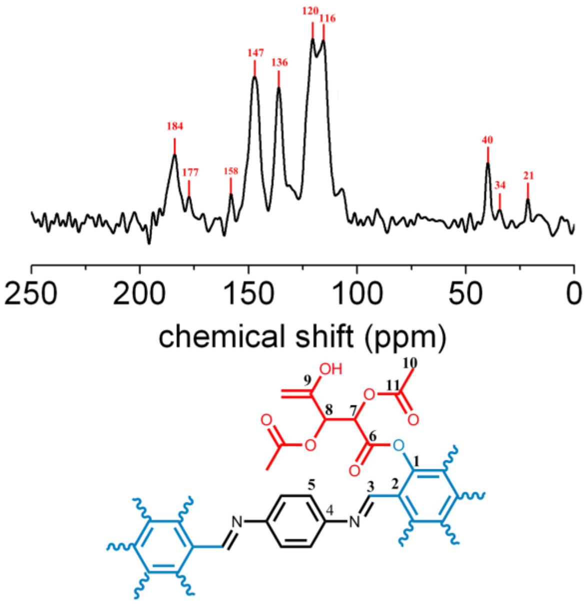

Carbon number	Chemical shift (ppm)
1	120
2	116
3	158
4	147
5	136
6	184
7	34
8	40
9	184
10	21
11	177

Supplementary Figure 8. Solid-state ^{13}C NMR spectra and peak assignment for CTpPa-1.

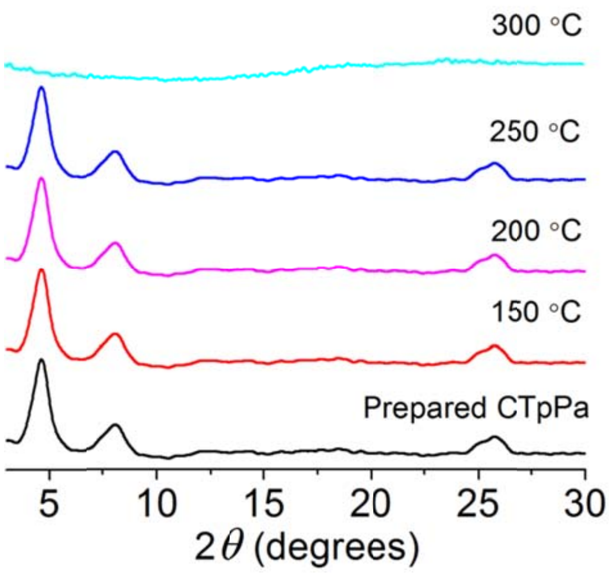

Supplementary Figure 9. PXRD patterns of CTpPa-1 treated at different temperatures under air for 1 h.

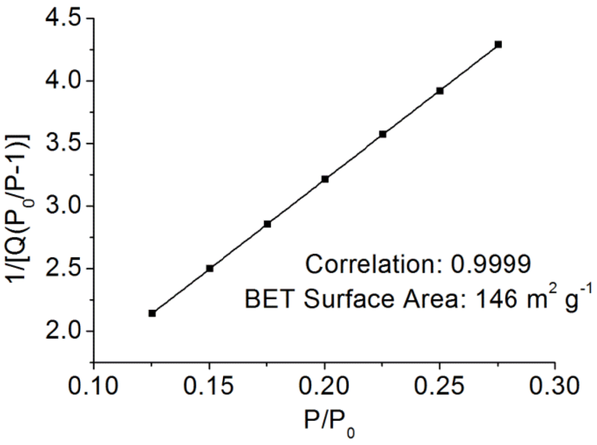

Supplementary Figure 10. BET surface area plot for CTpPa-1 calculated from the isotherm.

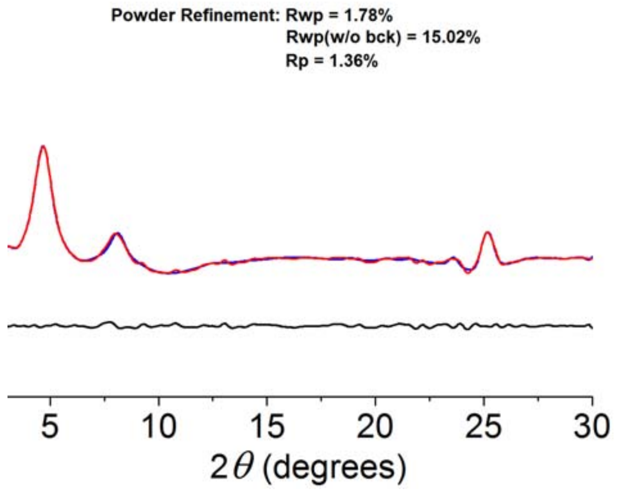

Supplementary Figure 11. Experimental PXRD pattern of CTpPa-2 (red curve), refined modeling PXRD pattern of CTpPa-2 (blue curve) and the difference plot of the two PXRD patterns (black curve).

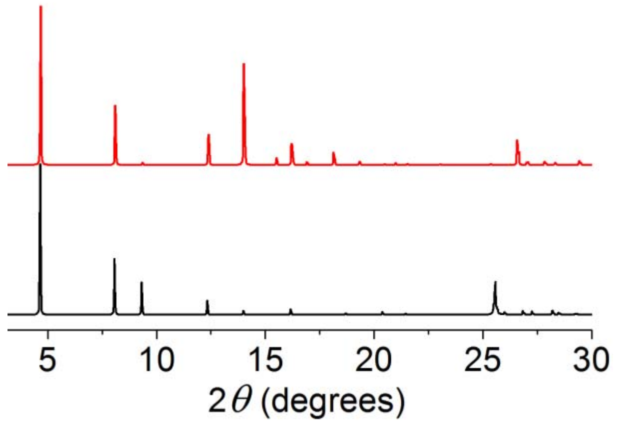

Supplementary Figure 12. Simulated PXRD pattern of CTpPa-2 for the eclipsed model (black curve) and the staggered model (red curve).

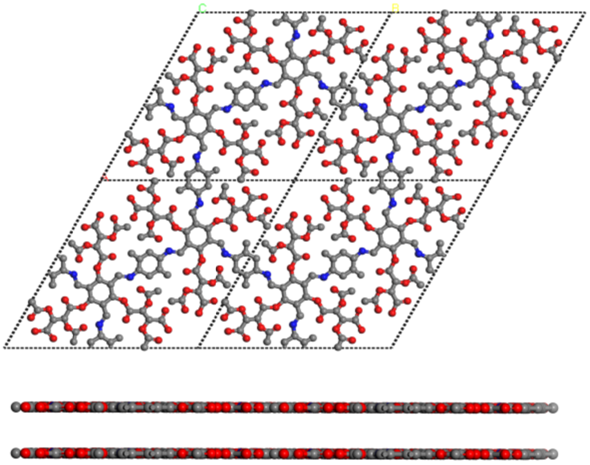

Supplementary Figure 13. Eclipsed structure of CTpPa-2. C, grey; N, blue; O, red, H is omitted for clarity.

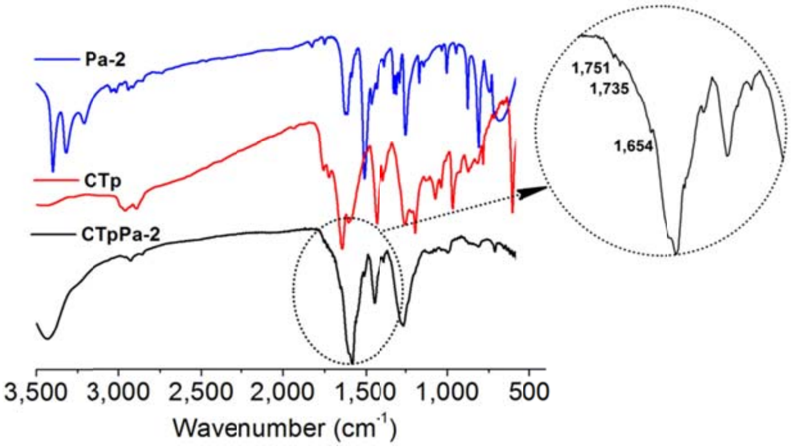

Supplementary Figure 14. FT-IR spectra of CTpPa-2, CTp, and Pa-2. The characteristic FT-IR peaks at 1751 cm^{-1} (C=O of ester), 1735 cm^{-1} (C=O of carboxyl) and 1654 cm^{-1} (C=N) for CTpPa-2 indicate that the successful formation of imine bonds and introduction of (+)-Ac-L-Ta.

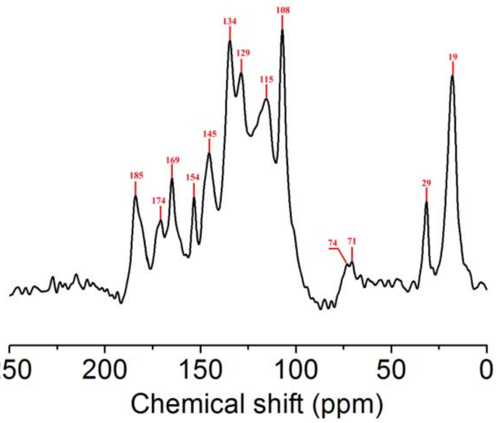

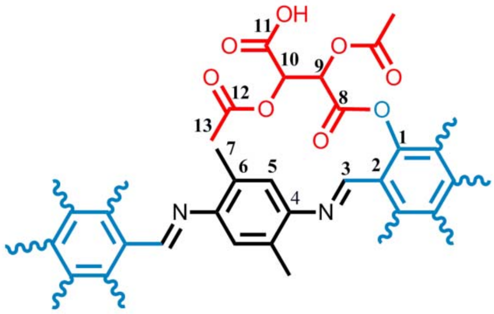

Carbon number	Chemical shift (ppm)
1	115
2	108
3	154
4	145
5	134
6	129
7	19
8	169
9	74
10	71
11	185
12	174
13	29

Supplementary Figure 15. Solid-state ^{13}C NMR spectra and peak assignment for CTpPa-2. The ^{13}C NMR chemical shift at 154 ppm is ascribed to the carbon atom of the C=N bond, which proves the formation of imine groups. Chemical shifts at 29, 74, 71, 169, 174 and 184 ppm are attributed to the carbon atoms of chiral ligand groups.

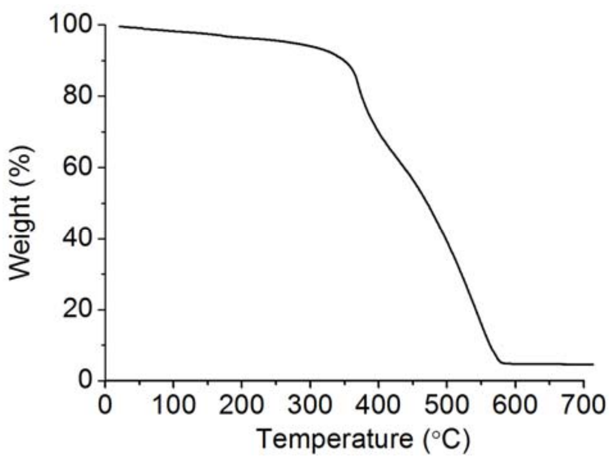

Supplementary Figure 16. TG curve of CTpPa-2. The TG curve shows no obvious weight loss until up to 350 °C.

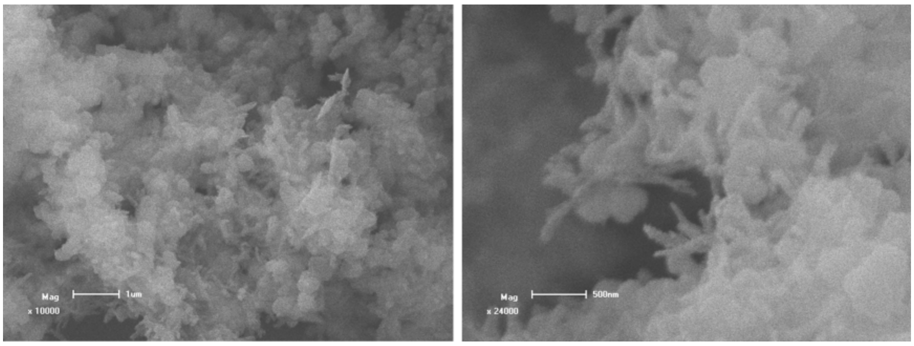

Supplementary Figure 17. SEM images of CTpPa-2.

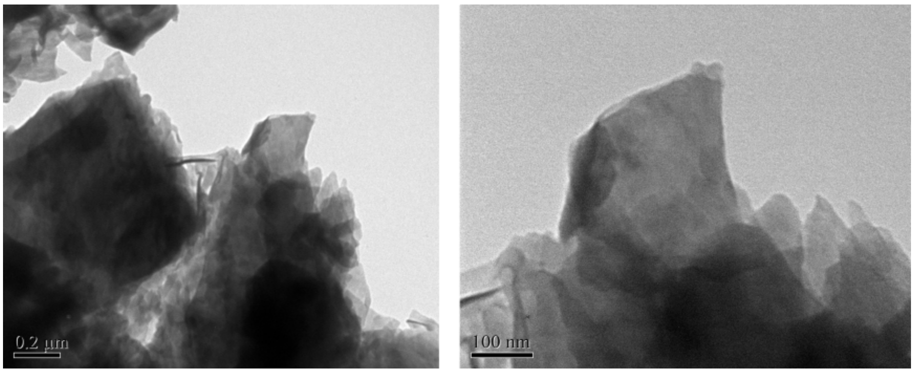

Supplementary Figure 18. TEM images of CTpPa-2.

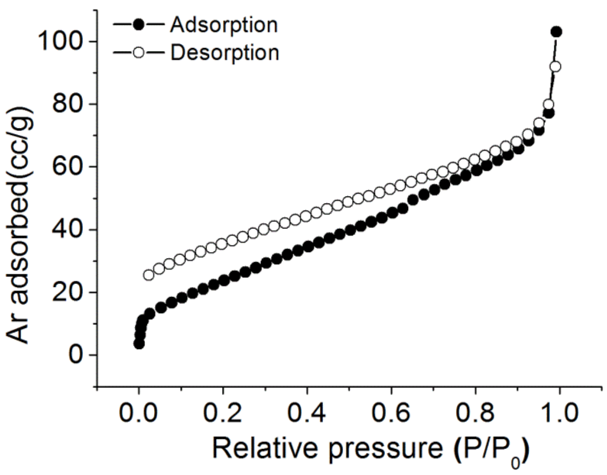

Supplementary Figure 19. Argon adsorption-desorption isotherms of CTpPa-2. The surface area and porosity of CTpPa-2, measured by Argon adsorption-desorption analysis, were 104 $m^2 g^{-1}$, 0.18 $cm^3 g^{-1}$, respectively.

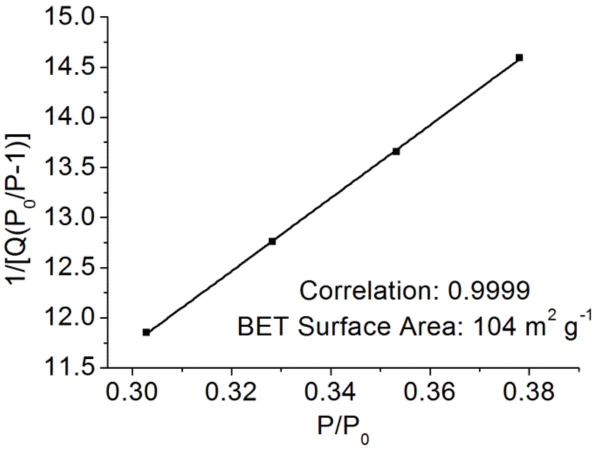

Supplementary Figure 20. BET surface area plot for CTpPa-2 calculated from the isotherm.

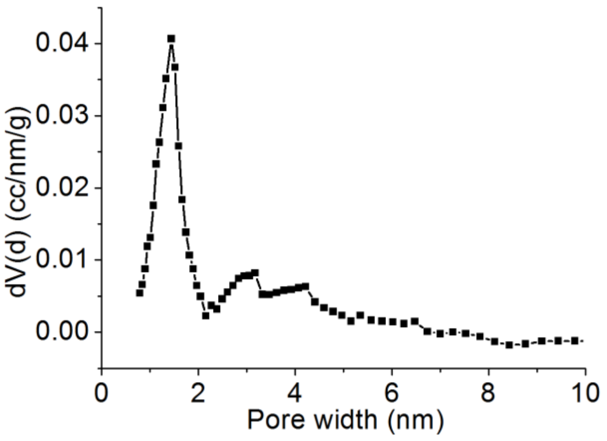

Supplementary Figure 21. Pore size distribution of CTpPa-2. The pore size of the CTpPa-2 calculated with nonlocal density functional theory was about 1.2 nm.

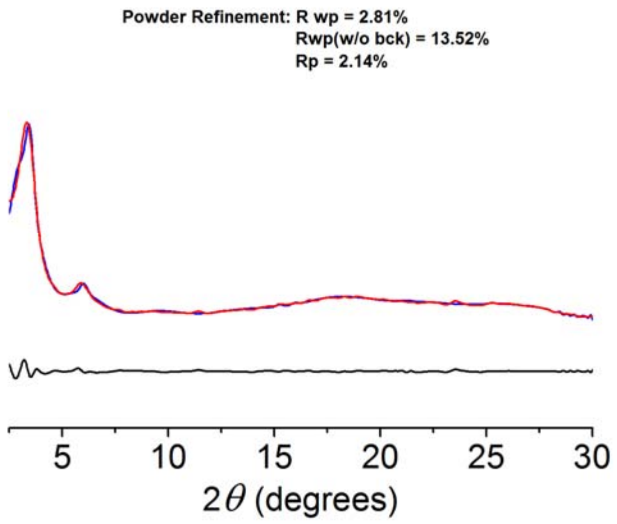

Supplementary Figure 22. Experimental PXRD pattern of CTpBD (red curve), refined modeling PXRD pattern of CTpBD (blue curve) and the difference plot of the two PXRD patterns (black curve)

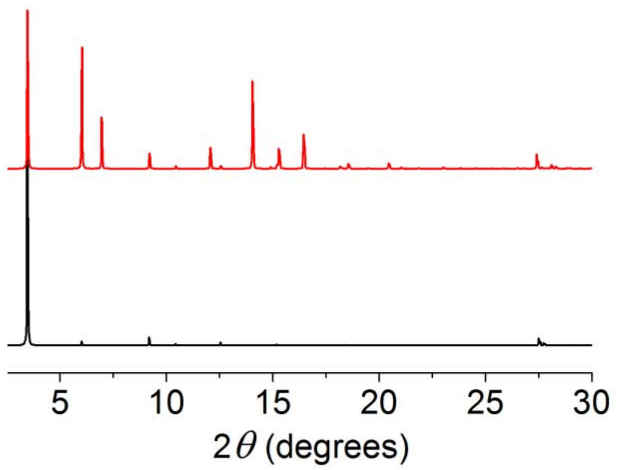

Supplementary Figure 23. Simulated PXRD pattern of CTpBD for the eclipsed model (black curve) and the staggered model (red curve).

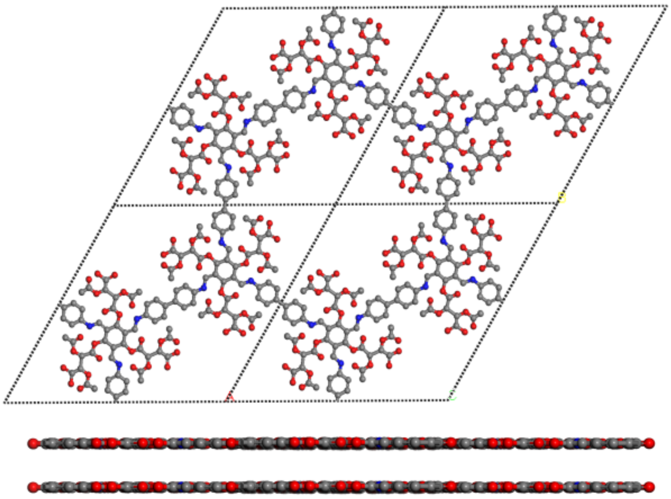

Supplementary Figure 24. Eclipsed structure of CTpBD. C, grey; N, blue; O, red, H is omitted for clarity.

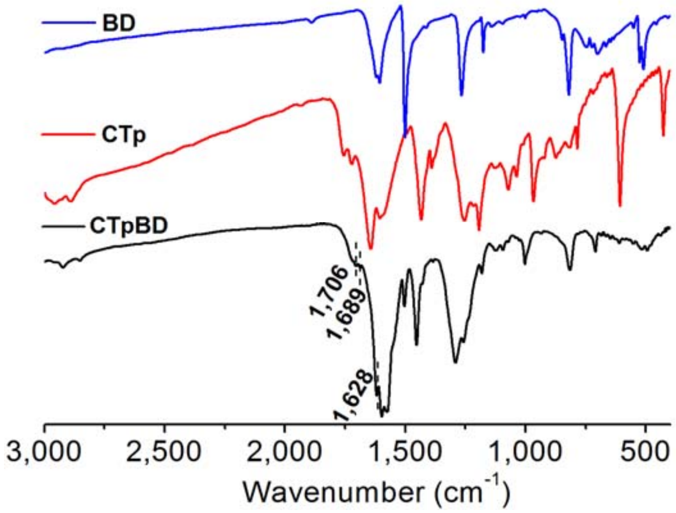

Supplementary Figure 25. FT-IR spectra of CTpBD, CTp, and BD. The characteristic FT-IR peaks at 1706 cm^{-1} (C=O of ester), 1689 cm^{-1} (C=O of carboxyl) and 1628 cm^{-1} (C=N) for CTpBD indicate that the the successful formation of imine bonds and introduction of (+)-Ac-L-Ta.

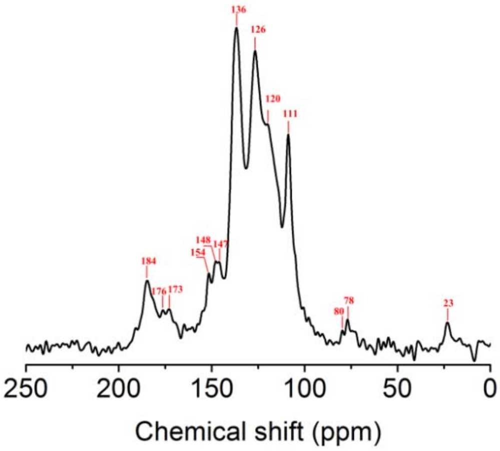

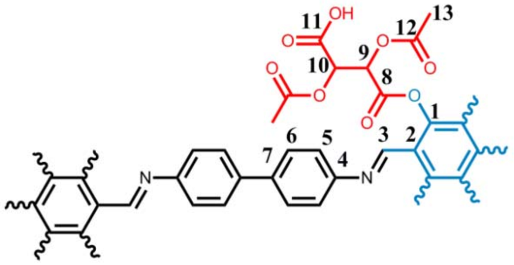

Carbon number	Chemical shift (ppm)
1	120
2	111
3	154
4	147
5	136
6	126
7	148
8	173
9	78
10	80
11	184
12	178
13	23

Supplementary Figure 26. Solid-state ^{13}C NMR spectra and peak assignment for CTpBD. The ^{13}C NMR chemical shift of CTpBD at 154 ppm is ascribed to the carbon atom of the C=N bond, which proves the formation of imine groups. Chemical shifts at 23, 78, 80, 173, 178 and 184 ppm are attributed to the carbon atoms of chiral ligand groups.

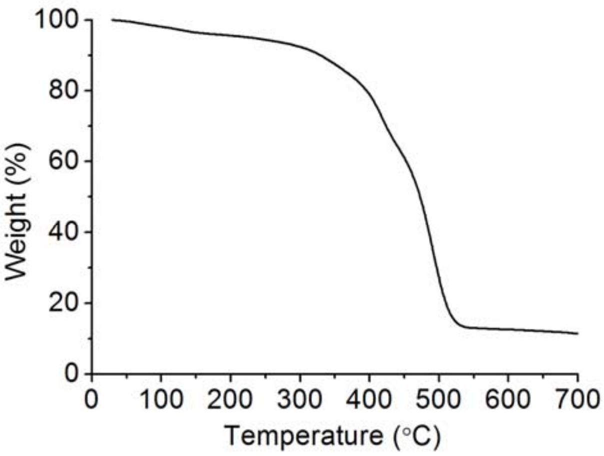

Supplementary Figure 27. TG curve of CTpBD. The TG curve shows no obvious weight loss until up to 300 °C.

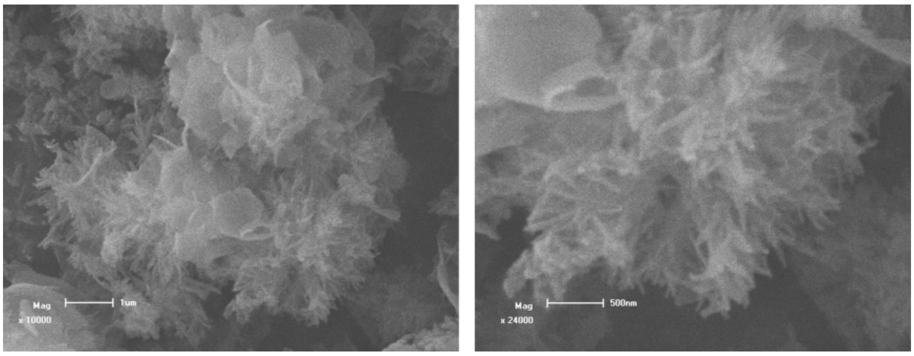

Supplementary Figure 28. SEM images of CTpBD.

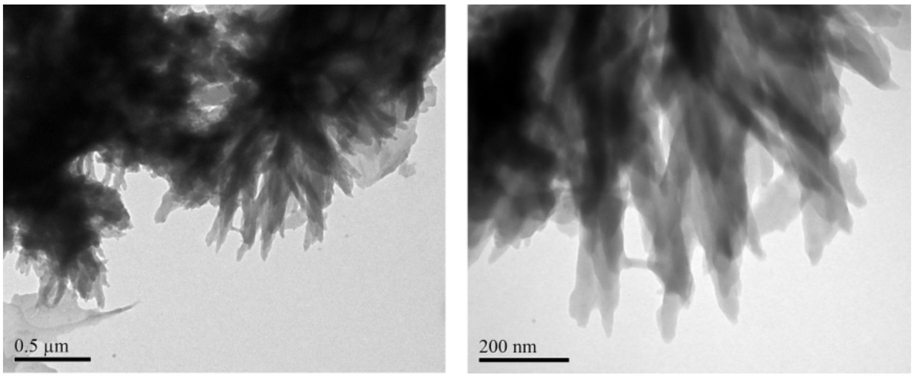

Supplementary Figure 29. TEM images of CTpBD.

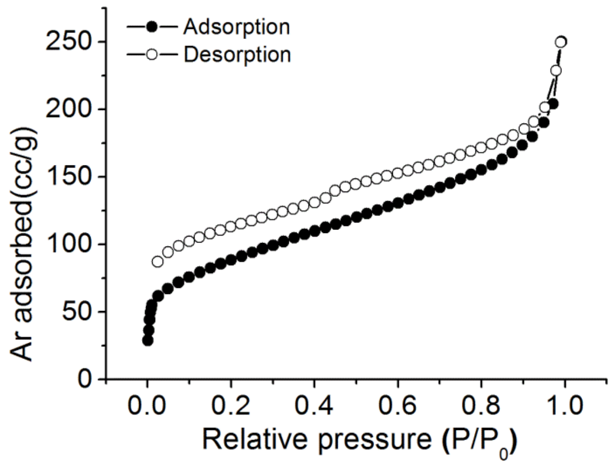

Supplementary Figure 30. Argon adsorption-desorption isotherms of CTpBD. The surface area and porosity of CTpBD, measured by Argon adsorption-desorption analysis, were 317 $m^2 g^{-1}$ and 0.30 $cm^3 g^{-1}$, respectively.

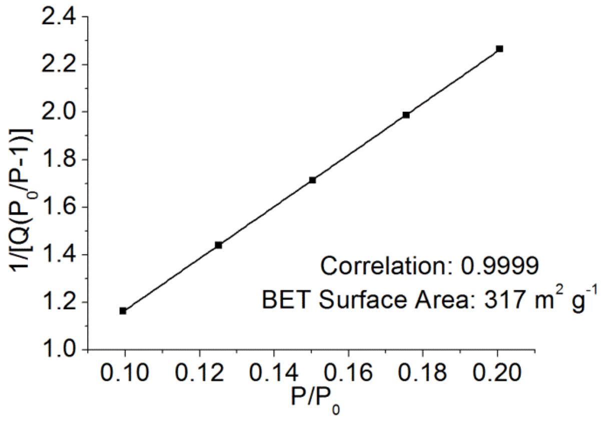

Supplementary Figure 31. BET surface area plot for CTpBD calculated from the isotherm.

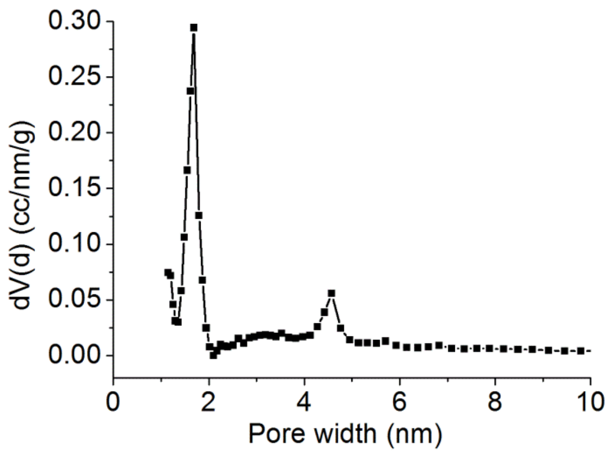

Supplementary Figure 32. Pore size distribution of CTpBD. The pore size of the CTpBD calculated with nonlocal density functional theory was about 1.8 nm.

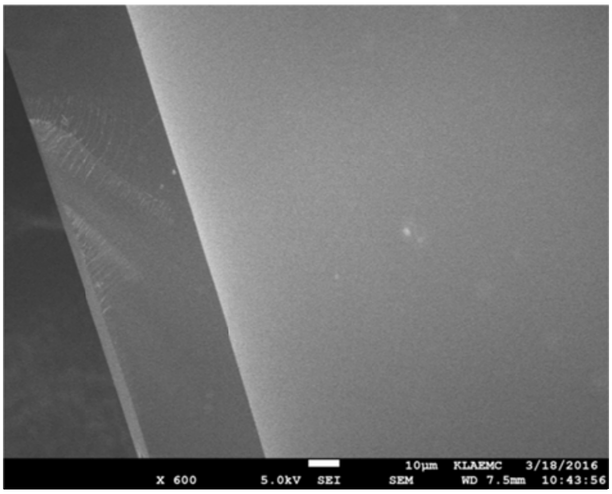

Supplementary Figure 33. SEM images of edge of dissected APTES modified capillary column.

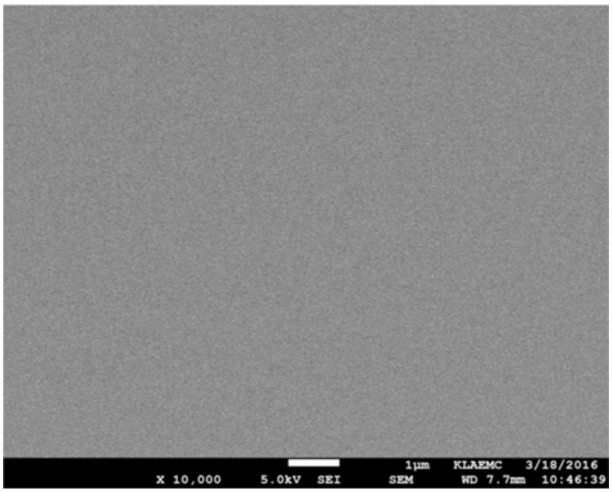

Supplementary Figure 34. SEM images of inner wall of APTES modified capillary column.

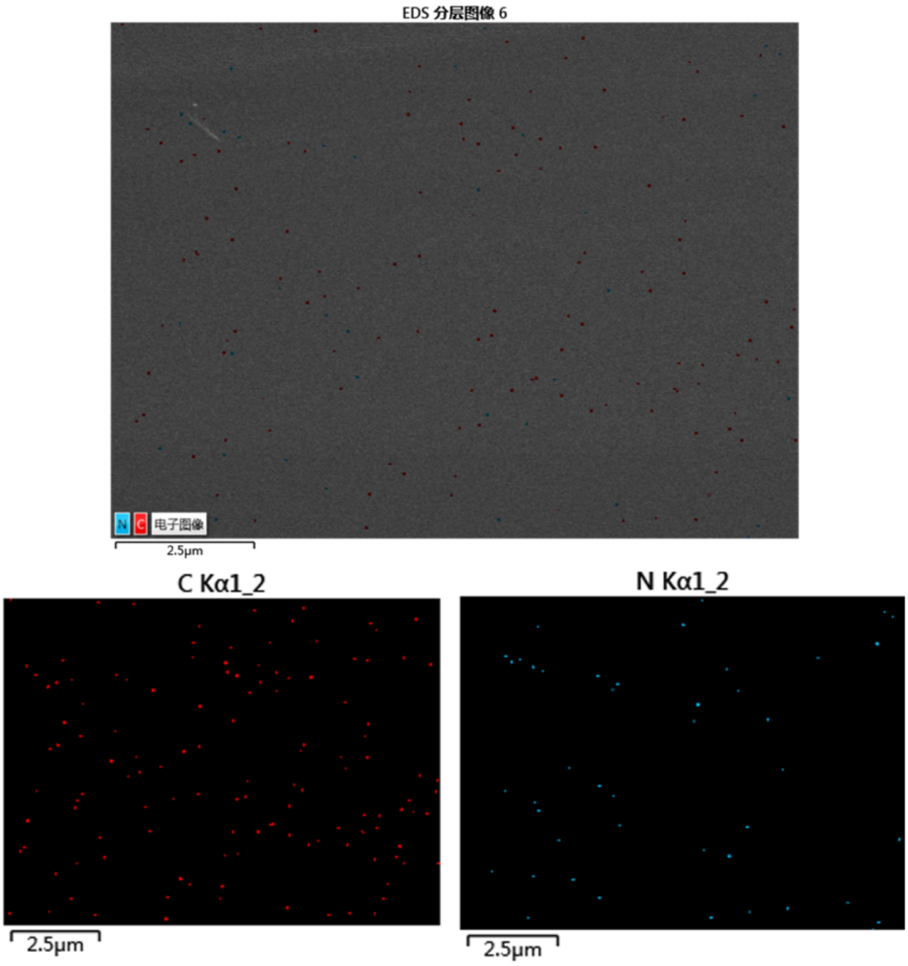

Supplementary Figure 35. EDS elemental mapping for the inner wall of APTES modified capillary column.

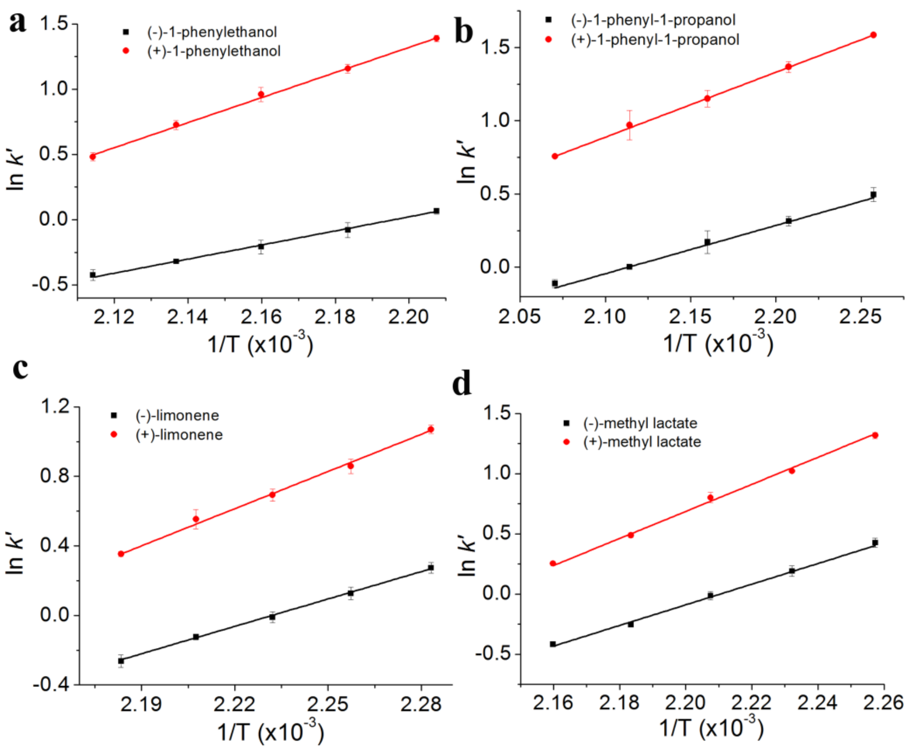

Supplementary Figure 36. Van't Hoff plots for the gas chromatographic separation of racemates on the CTpPa-1 bound capillary column.

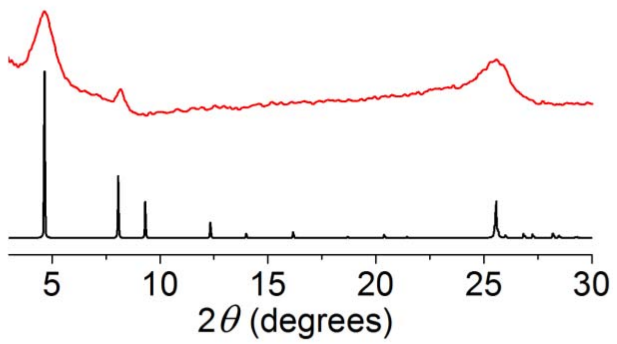

Supplementary Figure 37. PXRD pattern of the CTpPa-2 scraped from the fused-silica plate (red curve) and the simulated PXRD pattern of CTpPa-2 (black curve).

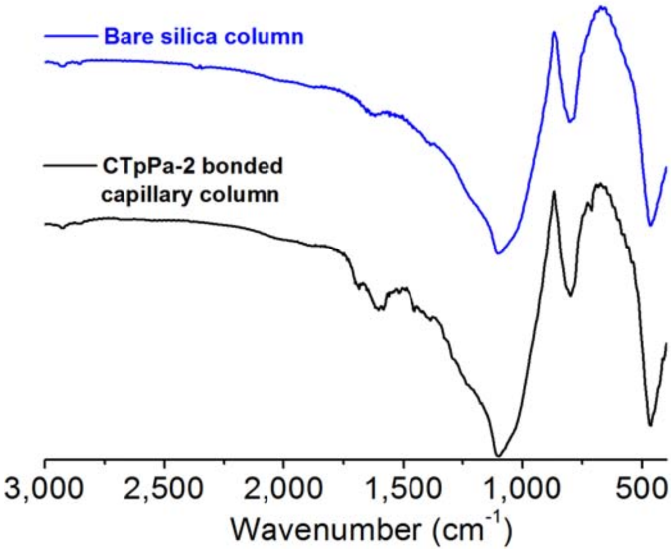

Supplementary Figure 38. FT-IR spectra of bare silica capillary column, CTpPa-2 bound capillary column. FT-IR peaks of the CTpPa-2 at 1658 cm^{-1} (C=N), and 1604 cm^{-1}, 1584 cm^{-1} (phenyl groups) in the CTpPa-2 bound capillary column reveals the successful bonding of CTpPa-2 in capillary column.

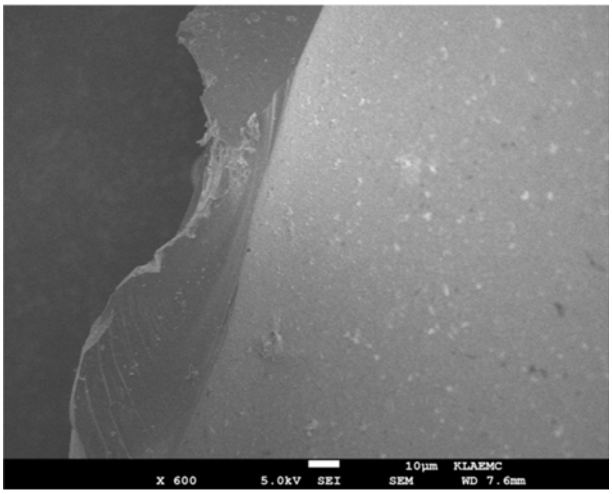

Supplementary Figure 39. SEM images of edge of dissected CTpPa-2 bound capillary column.

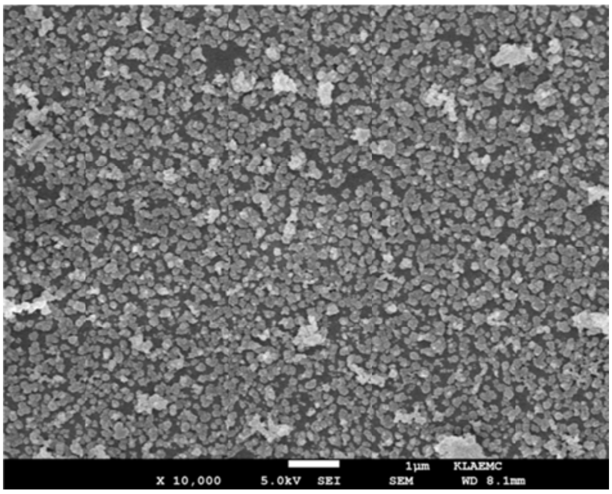

Supplementary Figure 40. SEM images of inner wall of CTpPa-2 bound capillary column.

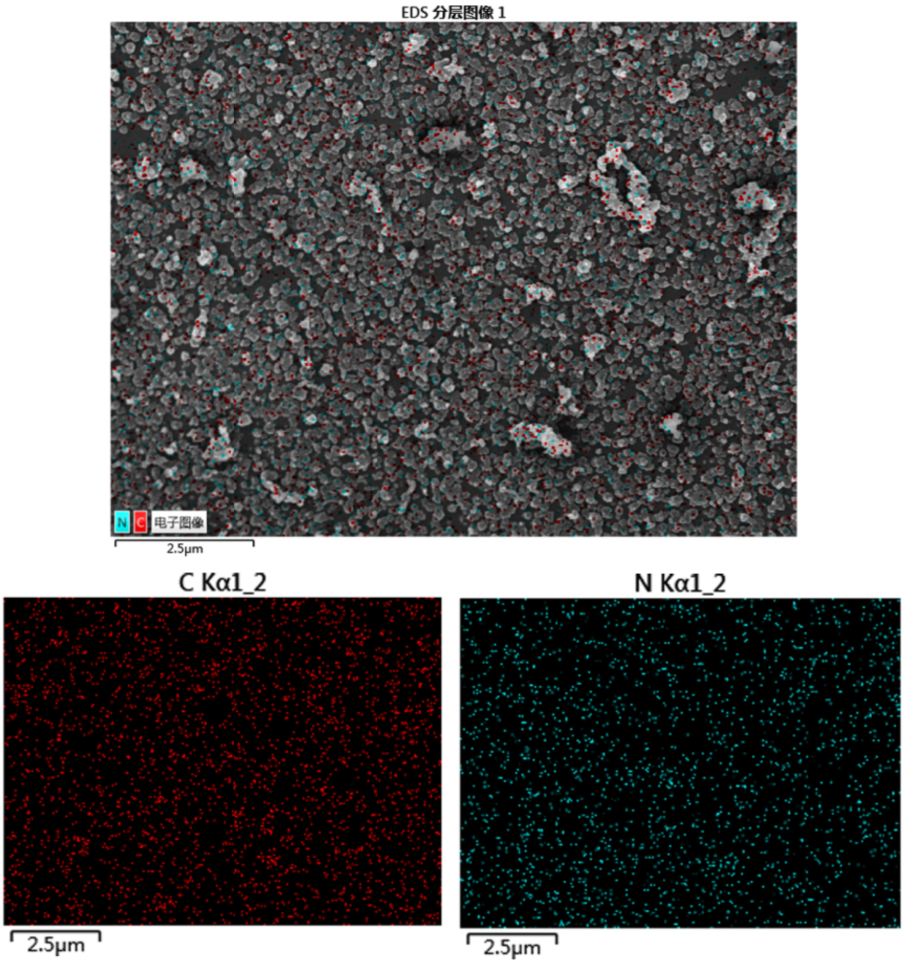

Supplementary Figure 41. EDS element mapping for CTpPa-2 bound capillary column.

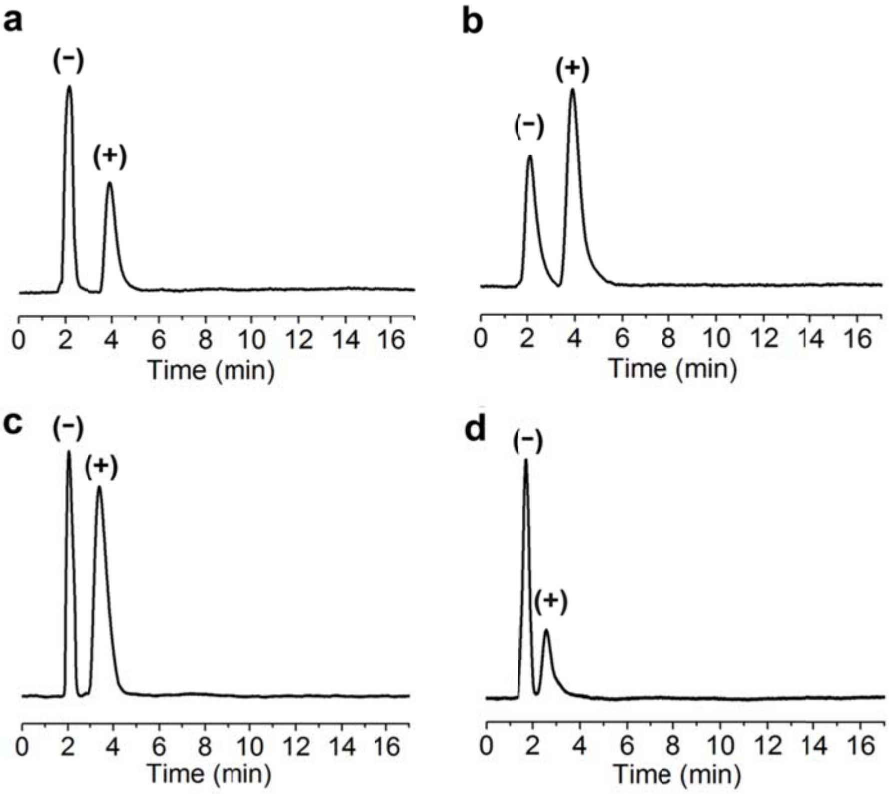

Supplementary Figure 42. Gas chromatograms for the separation of enantiomers on CTpPa-2 bound capillary column (30 m long × 0.32 mm i.d.). a, (+/-)-1-phenylethanol (200 °C, 1.5 mL min^{-1} N_2); b, (+/-)-1-phenyl-1-propanol (200 °C, 2 mL min^{-1} N_2); c, (+/-)-limonene (180 °C, 1.5 mL min^{-1} N_2); d, (+/-)- methyl lactate (170 °C, 1.5 mL min^{-1} N_2).

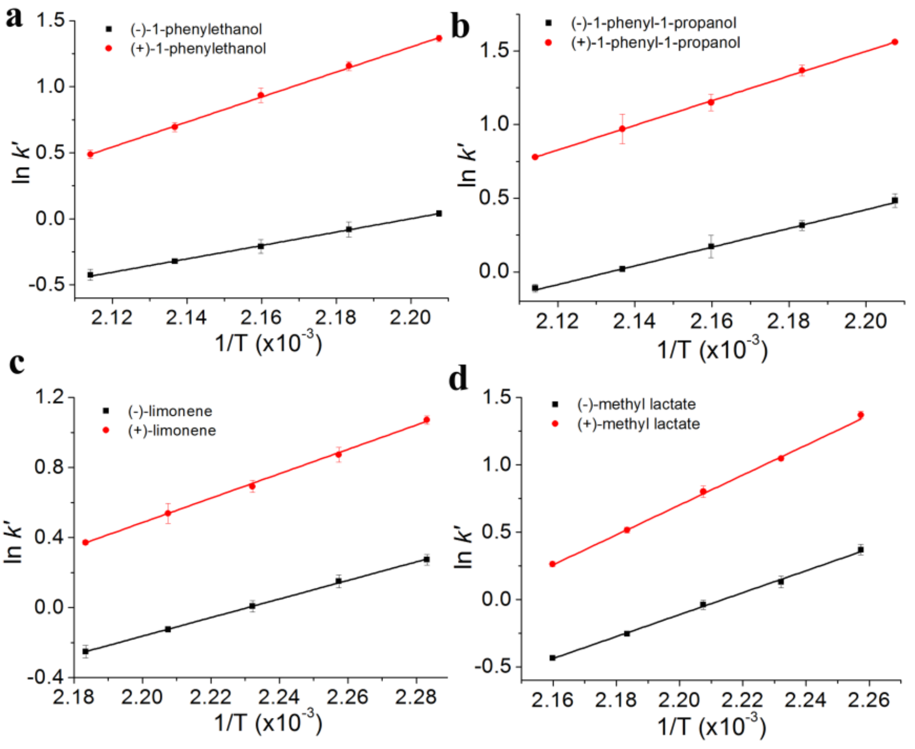

Supplementary Figure 43. Van't Hoff plots for the gas chromatographic separation of racemates on the CTpPa-2 bound capillary column.

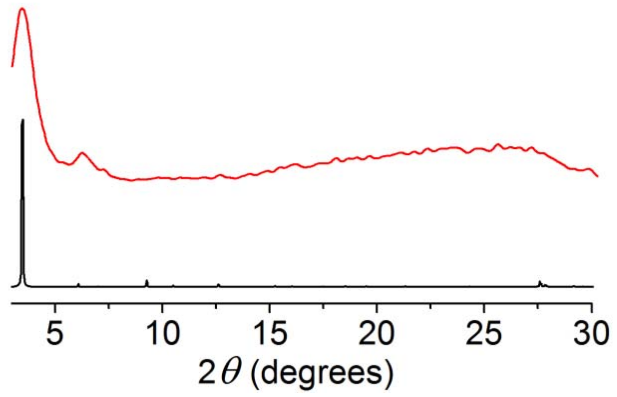

Supplementary Figure 44. PXRD pattern of the CTpBD scraped from the fused-silica plate (red curve) and the Simulated PXRD pattern of CTpBD (black curve).

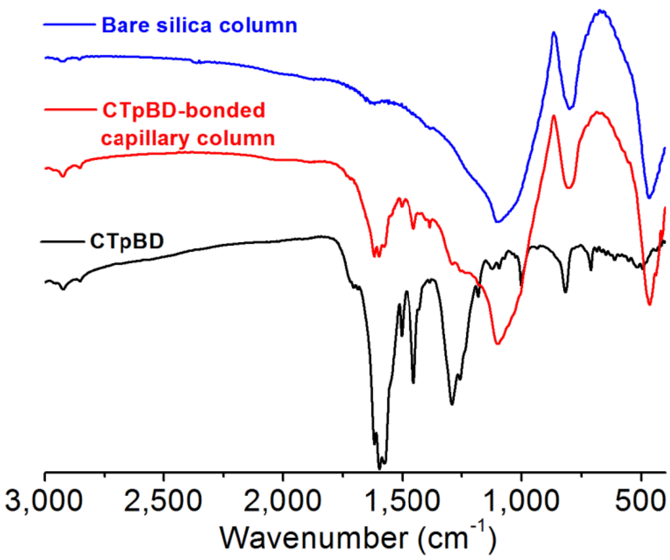

Supplementary Figure 45. FT-IR spectra of bare silica capillary column, CTpBD bound capillary column and CTpBD.

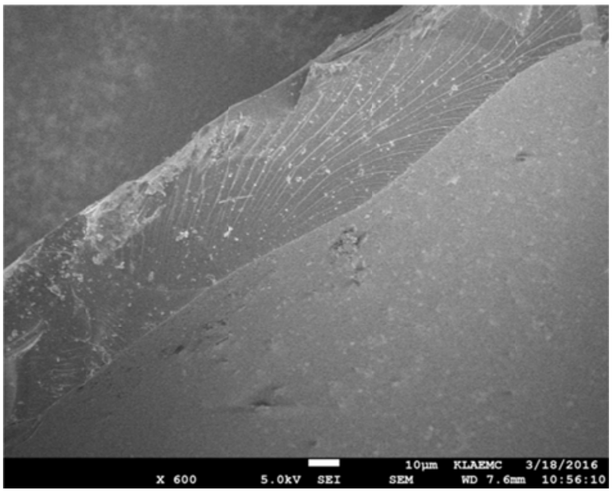

Supplementary Figure 46. SEM images of edge of dissected CTpBD bound capillary column.

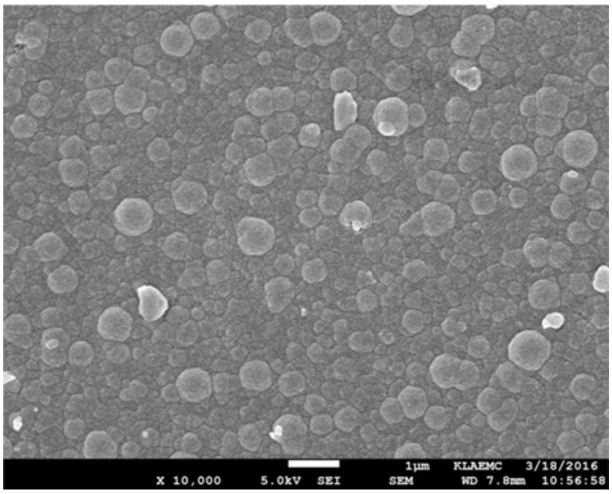

Supplementary Figure 47. SEM image of the inner wall of CTpBD bound capillary column.

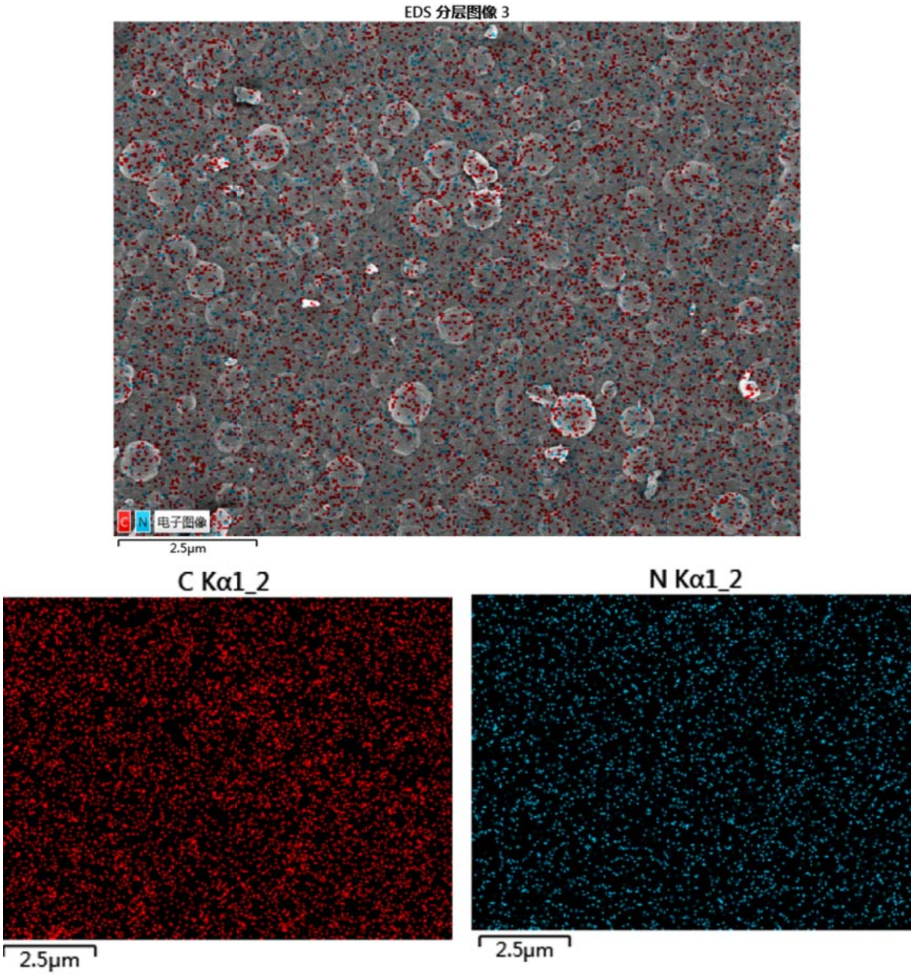

Supplementary Figure 48. EDS element mapping for CTpBD bound capillary column.

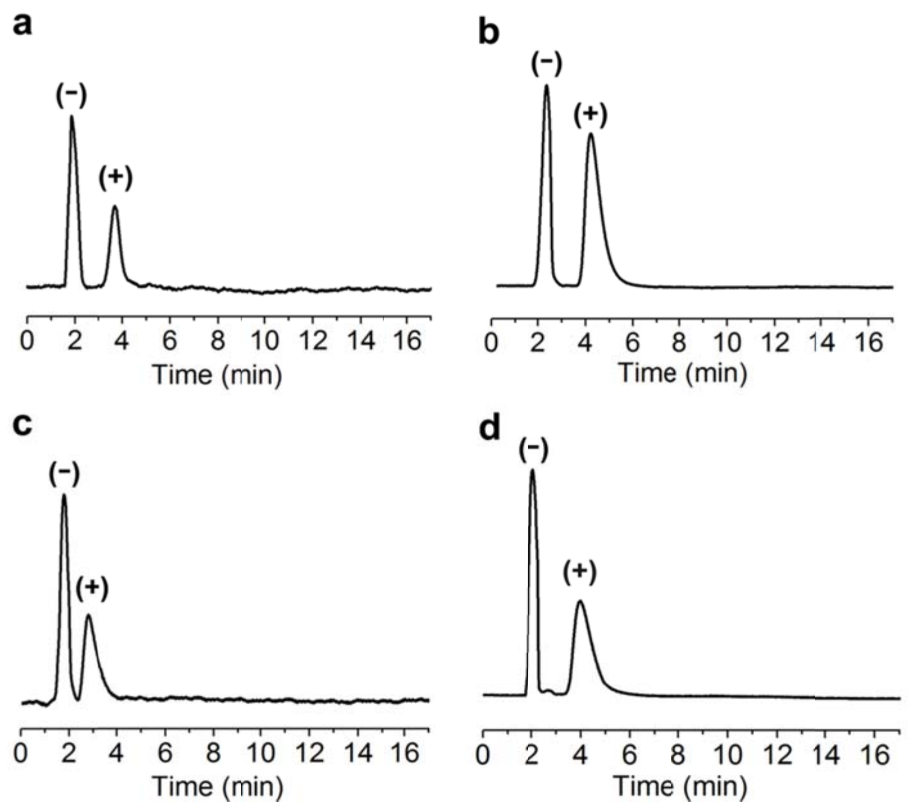

Supplementary Figure 49. Gas chromatograms for the separation of enantiomers on CTpBD bound capillary column (30 m long × 0.32 mm i.d.). a, (+/-)-1-phenylethanol (200 °C, 1.5 mL min^{-1} N_2); b, (+/-)-1-phenyl-1-propanol (200 °C, 2 mL min^{-1} N_2); c, (+/-)-limonene (180 °C, 1.5 mL min^{-1} N_2); d, (+/-)- methyl lactate (170 °C, 1.5 mL min^{-1} N_2).

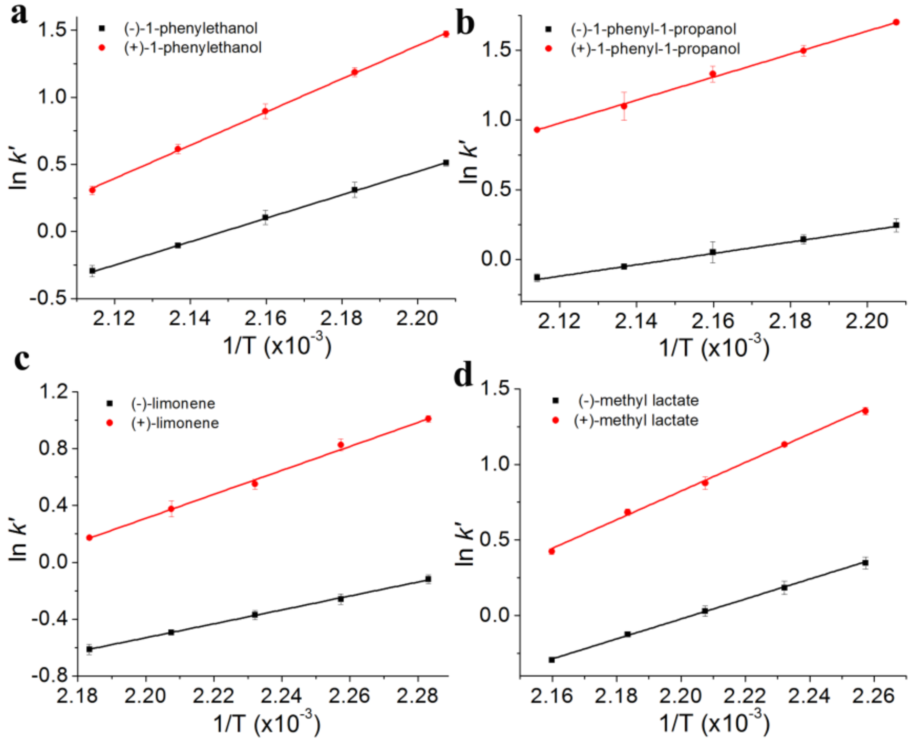

Supplementary Figure 50. Van't Hoff plots for the gas chromatographic separation of racemates on the CTpBD bound capillary column.

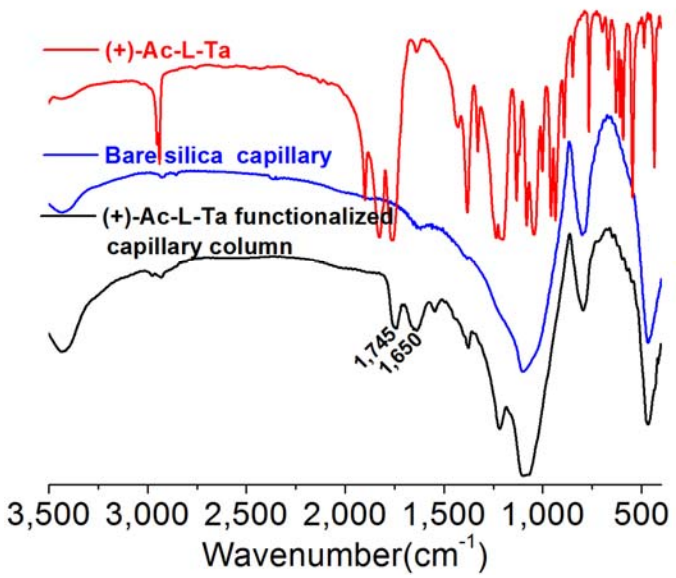

Supplementary Figure 51. FT-IR spectra of (+)-Ac-L-Ta, bare silica capillary column and (+)-Ac-L-Ta functionalized capillary column. The characteristic FT-IR peaks at

1745cm^{-1} (C=O of ester) and 1650 cm^{-1} (C=N) for CTpBD indicate that the the successful functionalization of (+)-Ac-L-Ta on GC column.

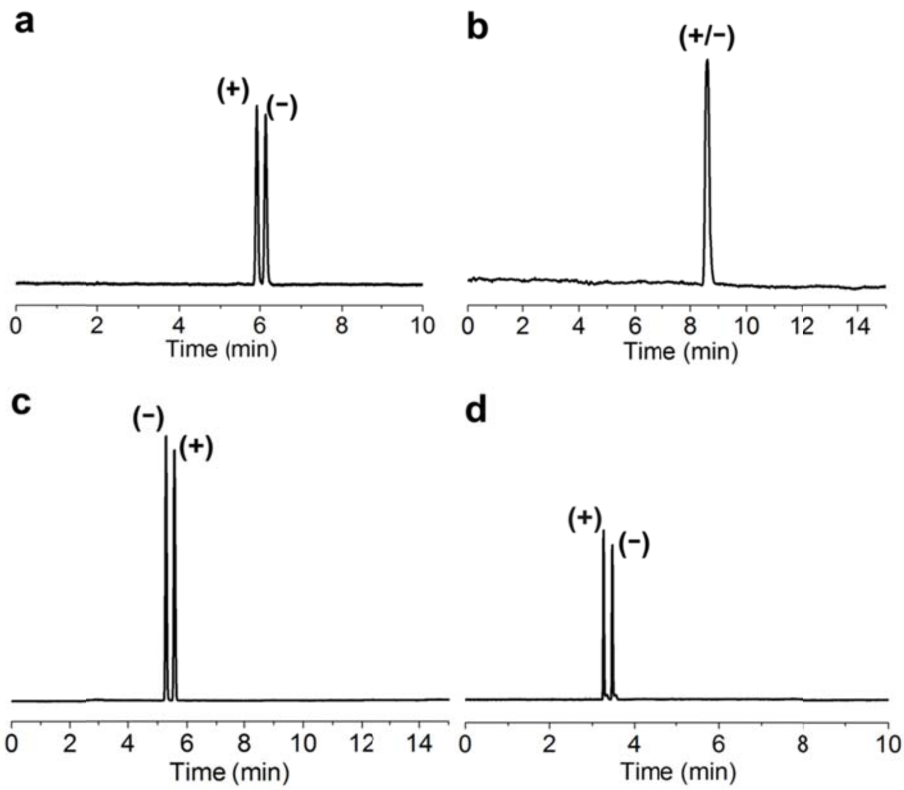

Supplementary Figure 52. Gas chromatograms for the separation of enantiomers on Cyclosil-B capillary column (30 m long × 0.32 mm i.d.). a, (+/-)-1-phenylethanol (130 °C, 2 mL min^{-1} N_2); b, (+/-)-1-phenyl-1-propanol (130 °C, 2 mL min^{-1} N_2); c, (+/-)-limonene (110 °C, 2 mL min^{-1} N_2); d, (+/-)- methyl lactate (100 °C, 1.5 mL min^{-1} N_2). Separation conditions were optimized to give the best separation of enantiomers.

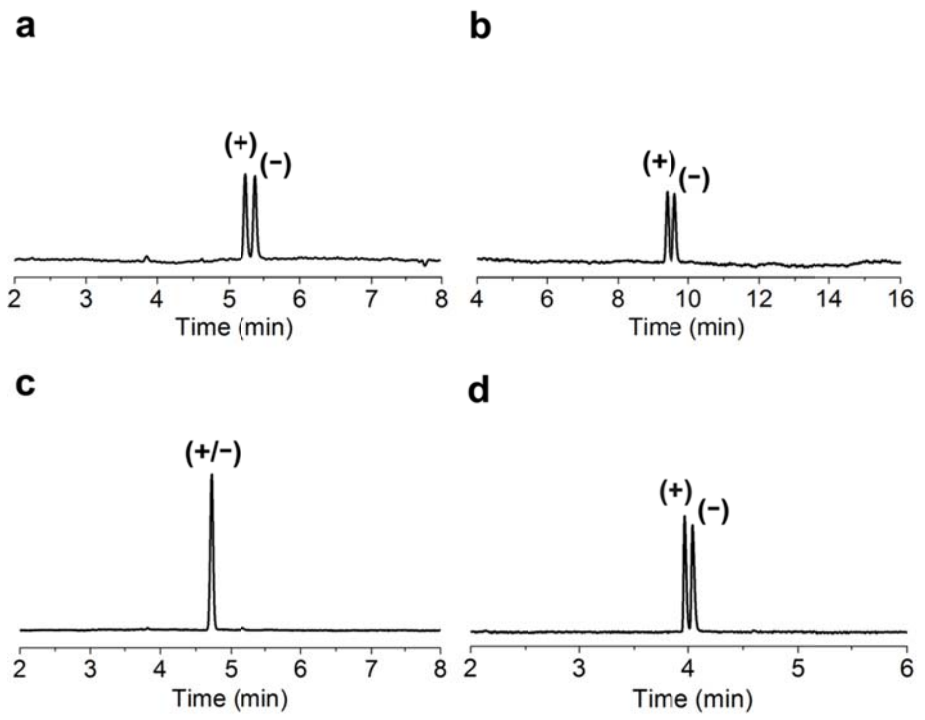

Supplementary Figure 53. Gas chromatograms for the separation of enantiomers on β-DEX 225 capillary column (30 m long × 0.25 mm i.d.). a, (+/-)-1-phenylethanol (130 °C, 2.0 mL min^{-1} N_2); b, (+/-)-1-phenyl-1-propanol (130 °C, 2 mL min^{-1} N_2); c, (+/-)-limonene (110 °C, 1.5 mL min^{-1} N_2); d, (+/-)- methyl lactate (130 °C, 2 mL min^{-1} N_2). Separation conditions were optimized to give the best separation of enantiomers.

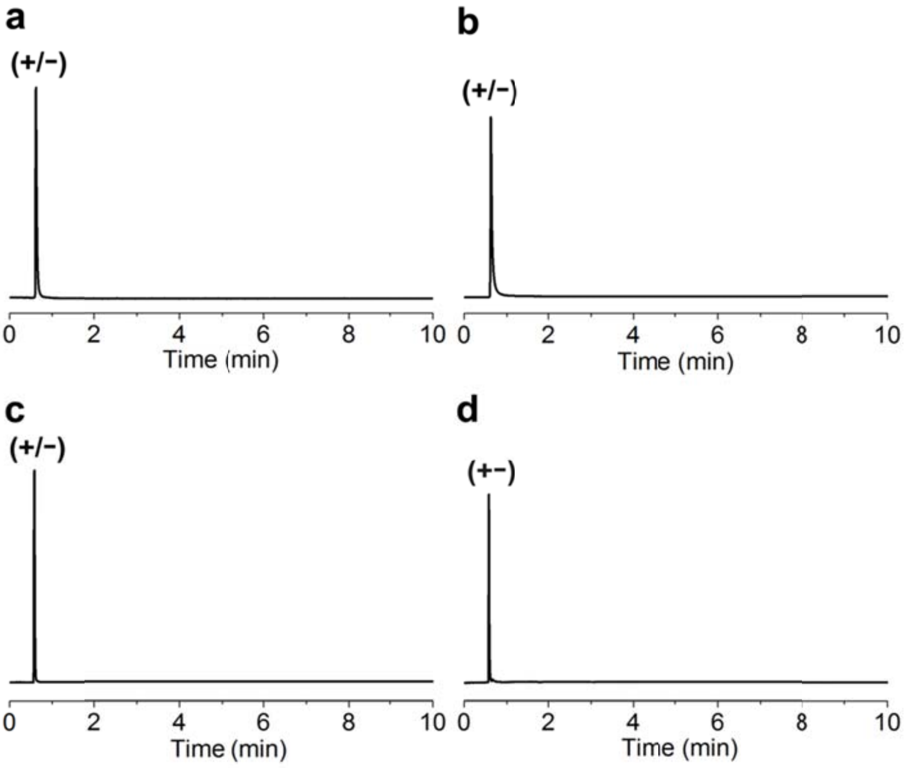

Supplementary Figure 54. Gas chromatograms for the separation of enantiomers on (+)-diacetyl-L-tartaric anhydride functionalized capillary column (30 m long × 0.32 mm i.d.). a, (+/-)-1-phenylethanol (150 °C, 2 mL min^{-1} N_2); b, (+/-)-1-phenyl-1-propanol (150 °C, 2.0 mL min^{-1} N_2); c, (+/-)-limonene (130 °C, 2 mL min^{-1} N_2); d, (+/-)- methyl lactate (120 °C, 2 mL min^{-1} N_2). No improvement of the separation was achieved by changing the separation temperature.

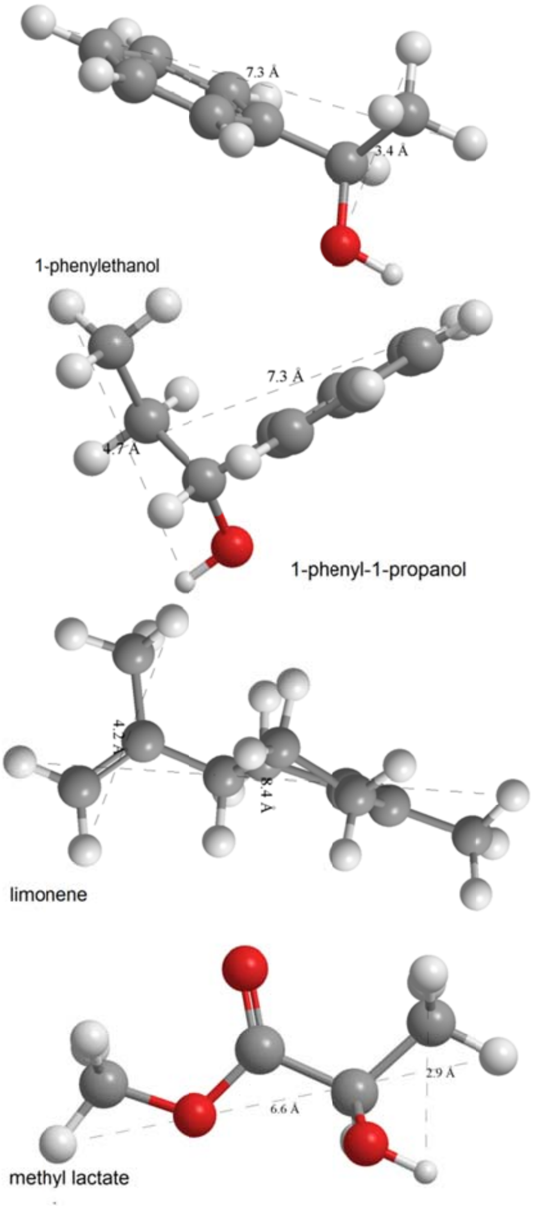

Supplementary Figure 55. The minimum kinetic diameter of 1-phenylethanol, 1-phenyl-1-propanol, limonene, methyl lactate (calculated with ChemBio3D).

Supplementary Table 1. Fractional main atomic coordinates for the unit cell of CTpPa-1 after Pawley refinement.

CTpPa-1: Space group symmetry P6/m<br>a = b = 21.9149 Å, c = 3.4824 Å, α = β = 90° and γ = 120°			
Atom	x	y	z
C1	0.73481	0.36776	0.50000
C2	0.70193	0.29699	0.50000
C3	0.80333	0.40877	0.50000
O4	0.73393	0.46708	0.50000
N5	0.85301	0.39944	0.50000
C6	0.92368	0.45639	0.50000
C7	0.96685	0.43307	0.50000
C8	0.95808	0.52798	0.50000
C9	0.73559	0.52629	0.50000
C10	0.79656	0.58570	0.50000
O11	0.67329	0.52273	0.50000
O12	0.85862	0.58879	0.50000
C13	0.79296	0.63909	0.50000
C14	0.85153	0.69416	0.50000
O15	0.73765	0.64335	0.50000
O16	0.90763	0.69278	0.50000
C17	0.85373	0.52595	0.50000
C18	0.74845	0.70438	0.50000
O19	0.80364	0.76072	0.50000
O20	0.80159	0.48282	0.50000
C21	0.90243	0.52155	0.50000
C22	0.68969	0.68855	0.50000
O23	0.85801	0.76002	0.50000

Supplementary Table 2. Fractional main atomic coordinates for the unit cell of CTpPa-2 after Pawley refinement.

CTpPa-2: Space group symmetry P6/m<br>a = b = 21.7511 Å, c = 3.5170 Å, $\alpha = \beta = 90°$ and $\gamma = 120°$			
Atom	x	y	z
C1	0.73933	0.35948	0.50000
C2	0.80652	0.37511	0.50000
O3	0.75807	0.47669	0.50000
N4	0.86081	0.43872	0.50000
C5	0.9287	0.46391	0.50000
C6	0.96476	0.43317	0.50000
C7	0.96461	0.53396	0.50000
C8	0.75943	0.5403	0.50000
C9	0.82442	0.60356	0.50000
O10	0.71326	0.54454	0.50000
O11	0.87875	0.61292	0.50000
C12	0.83259	0.66383	0.50000
C13	0.89488	0.72888	0.50000
O14	0.76888	0.6567	0.50000
O15	0.94317	0.71949	0.50000
C16	0.90486	0.5838	0.50000
C17	0.77406	0.71547	0.50000
O18	0.8192	0.77276	0.50000
O19	0.87775	0.52888	0.50000
C20	0.96252	0.62295	0.50000
C21	0.72615	0.72108	0.50000
O22	0.90048	0.79248	0.50000
C23	0.71212	0.40668	0.50000
C24	0.9398	0.37601	0.50000

Supplementary Table 3. Fractional main atomic coordinates for the unit cell of CTpBD after Pawley refinement.

CTpBD: Space group symmetry P6/m<br>a = b = 29.3557 Å, c = 3.2381 Å, $\alpha = \beta = 90°$ and $\gamma = 120°$			
Atom	x	y	z
O1	0.74115	0.44424	0.50000
C2	0.70116	0.38839	0.50000
C3	0.71952	0.35198	0.50000
N4	0.80945	0.42315	0.50000
C5	0.77453	0.37157	0.50000
C6	0.86282	0.4443	0.50000
C7	0.88577	0.41298	0.50000
C8	0.93904	0.43562	0.50000
C9	0.97167	0.48989	0.50000
C10	0.94782	0.52072	0.50000
C11	0.89425	0.49838	0.50000
C12	0.72999	0.47977	0.50000
C13	0.77023	0.53409	0.50000
O14	0.68769	0.46609	0.50000
O15	0.81924	0.5602	0.50000
C16	0.7438	0.57297	0.50000
C17	0.77097	0.62225	0.50000
O18	0.69312	0.55347	0.50000
O19	0.8174	0.64395	0.50000
C20	0.8454	0.53794	0.50000
C21	0.67423	0.58361	0.50000
O22	0.69556	0.62575	0.50000
O23	0.82522	0.4938	0.50000
C24	0.88977	0.56069	0.50000
C25	0.6307	0.56115	0.50000
O26	0.74911	0.65423	0.50000

Supplementary Table 4. Comparison of chiral separation ability of prepared chiral COF bound capillary columns with commercial chiral capillary columns

capillary column	(+/-)-1-phenylethanol		(+/-)-1-phenyl-1-propanol		(+/-)-limonene		(+/-)-methyl lactate	
	α	R	α	R	α	R	α	R
CTpPa-1	2.48	2.38	2.33	1.68	1.96	1.82	1.88	1.56
CTpPa-2	2.39	2.17	2.23	1.64	1.94	1.78	1.89	1.58
CTpBD	2.68	2.48	2.38	1.82	2.08	1.58	2.80	2.15
Cyclosil-B	1.04	1.40	1.00	0	1.07	1.67	1.06	2.14
β-DEX 225	1.05	1.78	1.04	1.48	1.00	0	1.02	1.34

α, separation factor, calculated according to $\alpha = t_2' / t_1'$, where t' is relative retention time.
R, resolution, calculated according to $R = 2(t_2-t_1) / (W_1+W_2)$, where t is retention time, W is peak width, the footnotes 1 and 2 refer to the first and second peaks of the enantiomers, respectively.

Cyclosil-B capillary column (30 m long × 0.32 mm i.d., Agilent Technologies).

β-DEX 225 capillary column (30 m long × 0.25 mm i.d., Supelco Inc.).

Supplementary Table 5. Thermodynamic parameters for the chiral separation of enantiomers on the CTpPa-1 bound capillary column

Analytes	Δ*H* (KJ mol^{-1})	Δ*S* (J mol^{-1} K^{-1})	ΔΔ*H* (KJ mol^{-1})	ΔΔS (J mol^{-1} K^{-1})
(-)-1-phenylethanol	-41.9 ± 1.9	-51.1 ± 1.8	-35.0 ± 2.4	-66.6 ± 3.2
(+)-1-phenylethanol	-76.9 ± 1.4	-117.7 ± 2.6		
(-)-1-phenyl-1-propanol	-52.4 ± 2.4	-70.8 ± 2.0	-16.7± 3.3	-28.0 ± 2.9
(+)-1-phenyl-1-propanol	-69.1 ± 2.2	-98.8 ± 2.1		
(-)-limonene	-42.5 ± 1.9	-53.8 ± 2.1	-13.3 ± 3.0	-24.2 ± 2.9
(+)-limonene	-55.8 ± 2.3	-78.1 ± 2.0		
(-)-methyl lactate	-67.9 ± 2.5	-109.2 ± 2.9	-25.3 ± 3.8	-49.4 ± 4.3
(+)-methyl lactate	-93.2 ± 2.8	-158.6 ± 3.3		

Supplementary Table 6. Thermodynamic parameters for the chiral separation of enantiomers on the CTpPa-2 bound capillary column

Analytes	Δ*H* (KJ mol^{-1})	Δ*S* (J mol^{-1} K^{-1})	ΔΔ*H* (KJ mol^{-1})	ΔΔS (J mol^{-1} K^{-1})
(-)-1-phenylethanol	-40.1 ± 2.4	-48.3 ± 2.1	-36.0 ± 3.3	-67.6 ± 3.8
(+)-1-phenylethanol	-76.1 ± 2.2	-115.9 ± 3.2		
(-)-1-phenyl-1-propanol	-50.9 ± 1.8	-67.8 ± 2.5	-16.4 ± 2.9	-27.3 ± 3.3
(+)-1-phenyl-1-propanol	-67.3 ± 2.3	-95.1± 2.2		
(-)-limonene	-42.5 ± 1.7	-53.8 ± 1.6	-13.2 ± 2.7	-23.8 ± 3.5
(+)-limonene	-55.7 ± 2.1	-77.6 ± 3.1		
(-)-methyl lactate	-65.5 ± 2.6	-103.9 ± 2.7	-24.7 ± 3.5	-48.0 ± 4.1
(+)-methyl lactate	-90.2 ± 2.3	-151.9 ± 3.1		

Supplementary Table 7. Thermodynamic parameters for the chiral separation of enantiomers on the CTpBD bound capillary column

Analytes	Δ*H* ($KJ\ mol^{-1}$)	Δ*S* ($J\ mol^{-1}\ K^{-1}$)	ΔΔ*H* ($KJ\ mol^{-1}$)	ΔΔS ($J\ mol^{-1}\ K^{-1}$)
(-)-1-phenylethanol	-69.3 ± 1.4	-107.9 ± 1.9	-30.2 ± 2.5	-58.4 ± 3.9
(+)-1-phenylethanol	-99.5 ± 2.1	-166.7 ± 3.4		
(-)-1-phenyl-1-propanol	-44.8 ± 1.9	-54.8 ± 2.3	-22.1 ± 2.9	-38.3 ± 3.4
(+)-1-phenyl-1-propanol	-66.9 ± 2.3	-93.1 ± 2.5		
(-)-limonene	-39.3 ± 1.5	-49.5 ± 1.2	-28.9 ± 2.1	-57.2 ± 2.2
(+)-limonene	-68.2 ± 1.4	-106.7 ± 1.9		
(-)-methyl lactate	-54.6 ± 2.2	-79.3 ± 2.5	-20.5 ± 3.3	-38.3 ± 3.7
(+)-methyl lactate	-75.1 ± 2.4	-117.6 ± 2.8		

Supplementary Table 8. Precision for the retention time of racemates on the CTpPa-1 bound capillary column

Analytes	RSD for retention time (%)		
	Run-to-run (n =7)	Day-to-day (n =5)	Column-to-column (n =3)
(-)-1-phenylethanol	0.27	1.11	3.41
(+)-1-phenylethanol	0.15	1.52	2.37
(-)-1-phenyl-1-propanol	0.24	1.69	3.15
(+)-1-phenyl-1-propanol	0.15	1.89	3.00
(-)-limonene	0.30	1.23	2.35
(+)-limonene	0.17	1.36	3.27
(-)-methyl lactate	0.22	1.21	2.69
(+)-methyl lactate	0.18	1.34	2.98

Supplementary Methods

Reagents. All chemicals and reagents used were at least of analytical grade. 1,3,5-Triformylphloroglucinol (Tp), 1,4-phenelynediamine (Pa-1), and benzidine (BD) were purchased from Aladdin Chemistry Co. Ltd. (Shanghai, China). (+)-Diacetyl-L-tartaric anhydride, cinene and 1-phenyl-1-propanol were purchased from J&K scientific Co. Ltd. (Beijing, China). 1-phenylethanol, 2,5-dimethyl-p-phenylenediamine (Pa-2) and methyl lactate were purchased from Tokyo chemical industry Co. Ltd. (Tokyo, Japan). Ethanol, methanol, 1,4-dioxane, mesitylene and tetrahydrofuran were obtained from Concord Chemical Research Institute (Tianjin, China) and dried through the standard procedures prior to use. Ultrapure water was obtained from Wahaha Foods Co. Ltd. (Tianjin, China). HCl, acetic acid and NaOH were purchased from Guangfu Fine Chemical Research Institute (Tianjin, China).

Instrumentation. ^{1}H nuclear magnetic resonance (NMR) spectra were recorded on a Bruker Avance III 400 MHz NMR spectrometer using tetramethylsilane (TMS) as an internal standard. Powder X-ray diffraction (PXRD) patterns were recorded on a D/max-2500 diffractometer (Rigaku, Japan) using Cu Kα radiation (λ = 1.5418 Å) with a scan speed of 8° min^{-1} and a step size of 0.02° in 2ϑ. Solid-state NMR experiments were performed on Infinityplus 300 (VARIAN, USA). Mass spectra (MS) experiment was carried out on Autoflex III TOF/TOF200 (Bruker, Germany). Thermogravimetric analysis (TGA) experiments were performed on a PTC-10A thermal gravimetric analyzer (Rigaku, Japan) under air from room temperature to 700 °C at a ramp rate of

5 °C min^{-1}. Scanning electron microscopy (SEM) images were recorded on a SS-550 (Shimadzu, Japan) and JSM-7500F (JEOL, Japan) scanning electron microscope. Transmission electron microscopy (TEM) was performed on a JEOL-100CXII microscope (JEOL, Japan). Fourier transform-infrared spectroscopy (FT-IR) spectra were measured on a Nicolet IR AVATAR-360 spectrometer (Nicolet, USA) with pure KBr pellets. Ar adsorption experiments were performed on an ASAP 2010 micropore physisorption analyzer (Micromeritics, Nor-cross, GA, USA) using argon adsorption at 77 K. The pore size distribution of prepared chiral COFs was calculated using the nonlocal density functional theory (NLDFT) method. Elemental analysis (EA) was carried out on a vario EL CUBE analyzer (Elementary, Germany). Gas chromatographic measurements were performed on a 7890A GC systerm (Agilent, USA) system with flame ionization detector (FID). Nitrogen (99.999%) was used as the carrier gas.

Synthesis of CTpPa-2 and CTpBD. CTp (0.1 mmol), Pa-2 or BD (0.15 mmol), 1,4-dioxane (10 mL) and mesitylene (10 mL) were mixed under ultrasonication (80 W, 10 min) to obtain a homogeneous dispersion and then transferred into a three-necked flask equipped with a condenser. The mixture was refluxed at 90 ^{o}C for 4 h with Ar protection. The obtained dark red colored precipitate was collected and washed with absolute 1,4-dioxane and mesitylene three times, then dried at 120 °C under vacuum for 24 h to get the CTpPa-2 or CTpBD in ca.70 % isolated yield. FT-IR (CTpPa-2, powder): 1751, 1735, 1654, 1601, 1583, 1557, 1509, 1448, 1265 cm^{-1}. Anal. Cald for $(C_{15}H_{14}NO_8)n$ (CTpPa-2): C 53.57; H 4.17; N 4.17. Found: C 51.84; H 4.07; N 4.10. PXRD (CTpPa-2, 2 theta): 4.7^{o}, 8.1^{o}, 25.2^{o}. FT-IR (CTpBD, powder): 1706, 1686,

1618, 1597, 1576, 1503, 1452, 1257, 823 cm^{-1}. Anal. Cald for $(C_{17}H_{14}NO_8)n$ (CTpBD): C 56.67; H 3.89; N 3.89. Found: C 55.35; H 3.58; N 3.64. PXRD (CTpBD, 2 theta): 3.4°, 6.0°, 27.5°.

Preparation of CTpPa-2 and CTpPD bound capillary columns. Solution A was obtained by suspending CTp (0.1 mmol) in 1,4-dioxane (5 mL) and mesitylene (5 mL), while solution B was obtained by dissolving Pa or BD (0.15 mmol) in 1,4-dioxane (5 mL) and mesitylene (5 mL). The above two solutions were cooled in an ice bath, and then mixed to obtain the pre-polymerization solution of CTpPa-2 or CTpBD. The amino-modified capillary was quickly filled with the pre-polymerization solution with a syringe and incubated in a 90 °C water bath for 4 h after both ends of the capillary were sealed with rubbers. The prepared capillary column was rinsed with 1,4-dioxane and mesitylene to remove residuals, then flushed with N_2 for 2 h to remove the solvent. Finally, the prepared corresponding chiral COFs bound capillary columns were conditioned with a temperature program: 80 °C for 30 °C min, ramp from 80 °C to 200 °C at a rate of 2 °C min^{-1}, and 200 °C for 1 h.

Preparation of (+)-diacetyl-L-tartaric anhydride functionalized capillary column. (+)-diacetyl-L-tartaric anhydride (1.2 mol) was dissolved in 20 mL THF, and then was injected into the amino-modified capillary. After incubation in a 60 °C water bath for 12 h, the prepared capillary column was rinsed with THF to remove the residuals, then flushed with N_2 for 2 h to remove the solvent. Finally, the prepared capillary column was conditioned with a temperature program: 80 °C for 30 °C min, ramp from 80 °C to 160 °C at a rate of 2 °C min^{-1}, and 160 °C for 1 h.

Calculation of thermodynamic parameters. The enthalpy change (ΔH) and entropy change (ΔS) for the transfer of the analyte between the mobile phase and the stationary phase were calculated according to the van't Hoff equation[1]:

$$\ln k' = -(\Delta H / R)\, 1/T + (\Delta S / R + \ln \Phi) \quad (1)$$

where k' is retention factor, R is gas constant, T is absolute temperature, and Φ is the phase ratio, which is defined as the volume ratio of the stationary phase (V_s) to the mobile phase (V_m).

The chiral part of enthalpy change ($\Delta\Delta H$) and entropy change ($\Delta\Delta S$) of the enantiomer–selector phase transfer were calculated as $\Delta H_2 - \Delta H_1$ and $\Delta S_2 - \Delta S_1$ for the enantiomers, respectively, where the footnotes 1 and 2 refer to the first and second peaks of the enantiomers, respectively.

Structural simulation and PXRD analysis. Molecular modeling of the CTpPa-1 was performed with Material Studio (ver. 7.0) suite of programs by Accelrys and referred to the method of previous work[2]. The unit cell was defined by two CTp molecules bound to one Pa via six hydrazine linkages. The initial structure was geometry optimized using MS Forcite molecular module (Universal force fields, Ewald summations). Due to the supposed structure of CTpPa-1 similar to the COF-LZU1[3], the initial lattice was generated with the space group P6/*m*, with a = b = 22.04 Å, c = 3.73 Å, and optimized using MS Forcite molecular module. Subsequently, pawley refinement was applied to refine the lattice parameters, producing the refined PXRD profile with lattice parameters a = b = 21.9149 Å, c = 3.4824 Å, Rwp = 1.73%, Rp = 1.35%.

Molecular modeling of CTpPa-2 and CTpBD was performed in the same way as for CTpPa-1. The results imply that the structure of CTpPa-2 adopts the AA stacking mode of a space group P6/m with a = b = 21.7511 Å, c = 3.5170 Å, $\alpha = \beta = 90°$ and $\gamma = 120°$ while the structure of CTpBD adopts the AA stacking mode of a space group P6/m with a = b = 29.3557 Å, c = 3.2381 Å, $\alpha = \beta = 90°$ and $\gamma = 120°$.

Supplementary References

1. Anderson, J. L. & Armstrong, D. W. Immobilized ionic liquids as high-selectivity/high-temperature/high-stability gas chromatography stationary phases. *Anal. Chem.* **77**, 6453-6462 (2005).
2. Ding, S. Y., Gao, J., Wang, Q., Zhang, Y., Song, W. G., et al. Construction of covalent organic framework for catalysis: Pd/COF-LZU1 in Suzuki-Miyaura coupling reaction. *J. Am. Chem. Soc.* **133**, 19816-19822 (2011).
3. Stegbauer, L., Schwinghammer, K. & Lotsch, B. V. A hydrazone-based covalent organic framework for photocatalytic hydrogen production. *Chem. Sci.* **5**, 2789-2793 (2014).
